# Breaking the silence: Understanding the impact of sexual violence on mental health in Tunisia

**DOI:** 10.1192/j.eurpsy.2025.2284

**Published:** 2025-08-26

**Authors:** K. Abdessattar, B. Amamou, H. Ktari, H. Mami, M. Oumaya, R. Bouzid

**Affiliations:** 1 Mental health, Med Taher Maamouri Hospital, Nabeul; 2 Mental health, Fatouma Bourguiba hospital, Monastir, Tunisia

## Abstract

**Introduction:**

Sexual violence is considered to be one of the most traumatic, pervasive, and common human rights violations. In Tunisia, there is limited research about this issue.

**Objectives:**

The present study aims to explore the relationship between sexual violence and mental health in Tunisia, with a focus on understanding the prevalence and types of mental health problems experienced by victims, as well as different factors related to it.

**Methods:**

This is a descriptive cross-sectional study that took place over six months from September 2022 to March 2023. Data collection was carried out using an online self-administered questionnaire distributed online. It is composed of 24 questions with “restricted” answers in the form of propositions. Each participant was invited to fill out the sociodemographic and clinical data form, the Harvard trauma questionnaire and the Hopkins symptoms questionnaire.

**Results:**

86.1% of our participants reported that they were subjected to a form of sexual aggression. Including 95.5% female, 4% male, and 0.5% non-binary. The median reported age of the first sexual aggression was 14 years. Our study found no significant correlation between age and trauma outcomes. Participants who reported sexual aggression were more likely to have scores above the cutoff for both the Harvard PTSD score and the Hopkins Symptoms Checklist scores. No statistically significant difference was found in the comparison of scores across genders. Participants with a past medical psychiatric history had a significantly higher average Harvard PTSD score as well as Hopkins symptoms checklist scores compared to those without this history. 35.5% of our participants chose not to disclose their traumatic experience to anyone. Notably, 34% of disclosures were made to friends and 22% to family members. The study found no statistically significant difference in the scores for post-traumatic stress disorder (PTSD), anxiety, or depression between participants who disclosed the assault and those who kept it a secret. The aggressor’s identity is mostly unknown (34.7%). Higher scores were reported by those who identified their partner as the aggressor. Our participants reported that they were victims of more than one episode of sexual assault in 67.3% of cases, with a mean age of revictimization of 19 years. A lower age of first sexual aggression was a significant risk factor for subsequent revictimization. 3.8% of our participants took legal action against their aggressor, and they had significantly higher average scores on all measured items and total scores. 30.8% of our participants seek psychiatric help. And the main reason for that would be a lack of awareness and knowledge.

**Conclusions:**

The cultural context of Tunisia, intricately woven into the fabric of our study, emphasizes the need for targeted and culturally sensitive approaches to addressing the aftermath of sexual violence.

**Disclosure of Interest:**

None Declared

